# Engineering biology in the face of uncertainty

**DOI:** 10.1098/rsfs.2023.0001

**Published:** 2023-06-09

**Authors:** William T. Sloan, Tania L. Gómez-Borraz

**Affiliations:** James Watt School of Engineering, University of Glasgow, Glasgow, UK

**Keywords:** modelling, engineering biology, microbial ecology, synthetic biology

## Abstract

Combining engineering and biology surely must be a route to delivering solutions to the world's most pressing problems in depleting resources, energy and the environment. Engineers and biologists have long recognized the power in coupling their disciplines and have evolved a healthy variety of approaches to realizing technologies. Yet recently, there has been a movement to narrow the remit of engineering biology. Its definition as ‘the application of engineering principles to the design of biological systems' ought to encompass a broad church. However, the emphasis is firmly on construction ‘…of novel biological devices and systems from standardized artificial parts’ within cells. Thus, engineering biology has become synonymous with synthetic biology, despite the many longstanding technologies that use natural microbial communities. The focus on the nuts and bolts of synthetic organisms may be deflecting attention from the significant challenge of delivering solutions at scale, which cuts across all engineering biology, synthetic and natural. Understanding, let alone controlling, every component of an engineered system is an unrealistic goal. To realize workable solutions in a timely manner we must develop systematic ways of engineering biology in the face of the uncertainties that are inherent in biological systems and that arise through lack of knowledge.

## 1. Discussion

The genesis of the concept of controlling biological systems is often attributed to Loeb [1]. He stood back from the reductionist paradigm that pervaded biology in the late nineteenth century and believed, and indeed demonstrated that empirically derived rules could be used to control generic biological mechanisms across multiple organisms based on environmental cues. He was interested in outputs and believed that biological complexity need not be totally unravelled to achieve them [2]. This was described as the ‘engineering ideal in biology’ by commentators [3]. Detractors were numerous and persistent in their demands to see the phenomena fractured into precise descriptions of their fundamental biochemical component parts [3]. While Loeb did influence the thinking of several influential biologists, reductionism continued to dominate most biological disciplines during the last century, and it has undoubtedly delivered fundamental understanding of biology. Advances in molecular biology and in genetic manipulation, in particular, fuelled renewed interest in control that ultimately led to synthetic biology. Despite synthetic biology often citing Loeb as inspiration, it has layered a reductionist perspective on top of the desire for control to deliver something subtly different from Loeb's more pragmatic engineering ideal. Synthetic biology assumes, or at least aspires to, a control from the molecule up and thus an absolute, deterministic knowledge of how changes made in the genome will affect an organism's response to the environment [4]. This is the ultimate reductionism and, as such, it is ambitious. Indeed, it is more ambitious than most established engineering disciplines in that, like the Feynman quote, ‘what I cannot create, I do not understand’, it equates engineering with perfect understanding.

At the same time as Loeb was advocating, and defending, his engineering ideal, engineers had got their hands on biology and were, albeit crudely, enacting it. Most notably, to help solve the prosaic problem of preventing pollution of our coasts and rivers from organic industrial and domestic wastewaters [5]. They engineered systems where complex microbial communities are forced to grow in biofilm structures by metabolizing the organic waste and producing CO_2_ and other less harmful molecules. The biofilms are engineered to be retained on fixed structures or to settle out of the water so that clean water can be discharged into the environment. A suite of biologically informed but necessarily empirical rules have allowed engineers to build wastewater treatment works all over the world and transform sewage treatment into one of the most economically and environmentally important biotechnologies. In this case, the necessity of delivering a timely solution to the pressing problem of urban squalor in the nineteenth century was the mother of invention. The empirical design rules were, and still are, far from perfect. Technologies sometimes fail for inexplicable reasons and the crudeness of the rules makes further invention slow, but the biotechnologies have saved countless lives. Other technologies, such as biorefineries [6] and bioremediation [7], have similarly benefited from engineering biology built on a combination of knowledge of the natural history of organisms and pragmatic empiricism.

Synthetic biology has promised to deliver solutions to the current environmental, energy and agricultural problems in high-profile reports and papers over the last two decades [8]. Genetically modified organisms certainly have had an enormous influence on a variety of fields. Take, for example, the production of recombinant proteins by genetically modified bacteria. These are already synthesizing vital drugs, like insulin. Moving from gene editing through to a workable technology is still, however, a major challenge that many potential synthetic biology solutions fail to overcome [9]. Even for the expression of a single protein, the interactions between the modified genetic code, environmental cues and the vagaries of the host cell throw up unanticipated problems that can take years of trial and error, rather than deep biological knowledge-based interventions, to overcome. The resulting organisms then typically operate in a small window of environmental conditions so that they need to be grown over short periods of time and cosseted in pristine, highly controlled conditions that are expensive to operate, and only make economic sense for these high-value products. As the gene circuits become more complicated the potential suite of interactions grows nonlinearly. For some of the world's most pressing problems like pollution control or conversion of waste into bioenergy, where organisms need to persist in open environments, synthetic biology solutions have yet to be successfully scaled-up.

So, for both the engineered consortia of natural organisms in open systems and for the current, successful, high-value synthetic biology technologies Loeb's engineering ideal of control has been achieved, but not through a complete understanding and/or standardization of every component in the biological system. There is a general consensus that for both open biological systems and highly constrained white biotechnologies that the control and the design processes need to be improved significantly if we are to accelerate innovation [9,10]. However, taking a dogmatic approach to delivering a solution to a complex problem has been shown in other fields, like software engineering, to slow progress and increase the likelihood of failure [11]. The reductive vision of synthetic biology has its place but should not be allowed to obscure a broader, more pragmatic, perspective on engineering biology.

All engineering design from the construction of enormous infrastructure, like skyscrapers, bridges and dams to the smallest computer chips relies on mathematical models. Engineering biology is no different and more control will be delivered by improved models. However, improving a model for design does not necessarily occur by adding complexity. Take for example the design of a water distribution system to a large city [12]. These were being designed and implemented in the late nineteenth century when the most comprehensive description of fluid flow was the Navier–Stokes equations coupled to a turbulence model. Yet no engineer deployed the Navier–Stokes equations in design at the time; they could neither parameterize nor solve them. Rather they relied then, and still do today, on Bernoulli's equation; an elegant simplification of the physics that eschews much of the complexity of momentum transfers and encapsulated the flow in terms of energy. Had we waited for Navier–Stokes to be useable our cities would have been without water until the 1970s. Deterministic mathematical modelling that integrates all of the biological component parts is the holy grail in terms of synthetic biology design, but it requires a level of knowledge that we do not currently have. The most comprehensive descriptions are genome-scale metabolic models, which certainly serve as excellent tools for generating hypothesis on the metabolism of organisms [13]. However, if reaction kinetics are included then parameterizing the models becomes an extremely daunting prospect and to include the spatial distribution and dynamics of molecules in the cell would yield a model so complex that it could not be validated using existing experimental methods. Thus, while this reductionist modelling is important in corroborating our understanding, for the models to be used effectively in a timely manner at least some components of the system will be gross approximations [9]. Even then these models are essentially of the internal cell biology of a single organism. To deliver solutions at scale then the biology beyond the cell wall has to be considered. In focusing the remit of engineering biology too keenly on the construction component of synthetic biology we may fail to reward efforts to quantify and predict the interactions of cells within and between populations and with the chemical and physical environment, all of which are vital to the design of any biotechnology [10]. Modelling the internal biology of every cell in massive populations of synthetic or natural microorganisms would be overwhelming even if it were computationally feasible, which it is not. So, like Bernoulli's hydraulic model, a simpler description of the biology is required that eschews much of the internal biology and, also like Bernoulli's hydraulic model, energy-based approaches have proved extremely useful. Thus, in engineering, for the microbial communities used in environmental applications, design is guided by theory based on chemical thermodynamics [14]. The growth and yield of a population of microbes growing on particular electron acceptors and donors are governed by the free energy in the redox reaction they exploit [15]. In individual-based modelling of microbial populations, the energy balance has been applied to the metabolism and division of individual cells such that the spatial distribution of cells in a complex consortia can be explicitly simulated [16,17]. The energy approach, while operating at scale, does treat the microbes as omnipresent catalysts, such that if chemical conditions are supportive then functional groups of organisms will thrive. And yet there is little consensus on how the diversity of functional groups and the resilience of the community, or key members in it, can be predicted, which should be vital considerations in design. Thus, for models at all resolutions from the intracellular to whole bioreactors, our incomplete knowledge of the biological systems that we try to engineer, whether synthetic or natural, is one of the main uncertainties in designing solutions that will work at scale. It is imperative that we improve models, but it is also crucial that we are pragmatic. Pragmatism is a hallmark of engineering where solutions need to be delivered on time, even when some of the fundamental science is still obscure. So, in structural engineering the deformation of materials like concrete in response to stress depends on the intricate exchange of forces between the fibres, aggregates and cements, the details of which are fascinating but obscure, therefore, in design they are captured in a single macroscopic property, Young's modulus, which all engineers can interpret. Hydraulic engineers designing everything from pipe networks to flood embankments use Manning's *n* to characterize the roughness of complex, heterogeneous surfaces and estimate energy dissipation. Although the tortuous flow of water through porous media may be explicitly characterized in expensive laboratory experiments, for design, engineers use an effective conductivity, which they have documented for different rocks and soils. There is a plethora of such macroscopic parameters used routinely to deliver engineering solutions. Their derivation and the equations they are deployed in are often as ingenious as any literal, reductionist, full description of the system. We need more ways of wrapping up unresolved biology in parameters like Young's modulus, Manning's *n* and effective conductivity in engineering biology if we are to deliver solutions quickly. Furthermore, if we are honest about the uncertainty in these parameters then engineering biologists can begin to adopt systematic methods of dealing with uncertainty, such as limit state design, that will define the bounds within which their technologies will safely operate. Engineers, unlike Feynman, by necessity routinely create things that they do not ‘fully’ understand.

Lack of knowledge is only one uncertainty that engineering biologists ought to confront. A recent review of the challenges facing engineering biology [9] acknowledged an issue that has perhaps not received the attention it deserves in engineering biology: ‘Currently, most synthetic biology projects work to design and construct a cell or strain and then put it to work, performing a task like biosynthesis or biosensing. We either just hope that mutation and selection will not act upon our ‘finished product’ once it is in operation, or in some cases, we design it as best as we can to reduce this chance [18]’. The efforts to design organisms that can resist evolution have, for example, involved the creation of stable chassis organisms with minimal genomes comprising what are considered essential genes that are shared by several wild-type strains. These chassis can then be used as stable platforms into which synthetic biological parts can be spliced. However, they have only ever been used in highly controlled laboratory environments. When exposed to fairly innocuous stresses that they might experience in real biotechnologies, such as starvation, it has been shown that the chassis organisms accumulate mutations equally as fast as wild-type organisms and up to 1000 times faster than the background mutation rate. Furthermore, the mutations were more likely to be deleterious in the chassis organism [19]. So, for application where populations of synthetic organisms need to be long lived or are exposed to environmental fluctuations or biological competition, at the very least, the effects of evolution should be built into the design process from the start. Building in feedbacks, such as kill switches [20] and more sophisticated circuits [21], can stop well-characterized detrimental mutations gaining a foothold, but anticipating evolutionary trajectories is almost impossible. One surprising idea that seems to run counter to the reductionist, control paradigm in synthetic biology, is to let evolution control the design of laboratory organisms, but it has been suggested that this would need a whole new ‘engineering theory of evolution’ [22]. It may be that exploring the natural biodiversity of microbes would reveal organisms that can already do components of the desired biological transformation and that using them in an engineered system would be a more prudent route to a solution. It would be a shame to see this equally valid approach to engineering biology sacrificed by adopting a restricted, synthetic biology, perspective on the field. For open systems, it is not only the uncertainty from evolution that engineering biologists must cope with. Random events such as immigration, emigration, deaths and births are manifest in a contribution to the dynamics of the abundance of species in the system [23]. So, species may be lost or gained, or their abundance amplified purely by chance. Our ability to quantify the risk of these processes derailing a biotechnology may make the difference between its widespread acceptance or ultimate failure. Again, simple engineering models with macroscopic parameters whose values can index typical behaviours and be used directly in the calculation of risk, such as the effective community size [23], are an imperative.

Environmental extremes, biogeography, evolution and our lack of knowledge of much of the detail of a biological system mean that engineering biology solutions will always be pursued in the face of uncertainties. These will never be totally eradiated, but they can be quantified and, in some cases, reduced. Engineering biology needs to find ways of delivering solutions that are robust and resilient within the bounds of expected variability. Loeb's original engineering ideal was about effecting control without necessarily resolving every aspect of the biology and achieving this means bringing to bear all of the most up-to-date science and mathematics. Synthetic biology and the tools it has developed have a huge role to play, but the idea that engineering biology solutions must involve genetically modified organisms is dangerously restrictive.

## Data Availability

No original data were presented or used in this discussion.
